# Dr. J. Franklin Barnes

**Published:** 1914-12

**Authors:** 


**Dr. J. Franklin Barnes,** Bellevue 1875, died October 28, at his home in Watkins, aged 61. He was writing at his desk when death came suddenly. He had been in bad health for five years.

